# Modelling conditions of storing quality commercial eggs

**DOI:** 10.1016/j.heliyon.2021.e07868

**Published:** 2021-08-25

**Authors:** Jacqueline Akelo Gogo, Benson Edwine Atitwa, Cyrus Ngari Gitonga, David Muchangi Mugo

**Affiliations:** a Department of Mathematics and Statistics, University of Embu, Kenya; b Department of Computing and Information Technology, University of Embu, Kenya

**Keywords:** Chicken egg, Experimental design, Fixed model, Mixed model, Variance component

## Abstract

Egg storage has been a problem due to ineffective methods subjecting many farmers and egg retailers to losses. These techniques include various models involving statistical analysis of the storage conditions on the egg quality. Apparent deficiencies of the information from the randomized complete block design model prompted this study. The study evaluated the effect of storage temperature at three levels (5 ^*°*^*C*, 19.5 ^*°*^*C*, 30 ^*°*^*C*) and duration at four levels (2^*nd*^, 12^*th*^, 22^*nd*^, 32^*nd*^) on egg quality using a fixed and mixed-effect model. We used a total of 618 fresh and unfertilized eggs from ISA (Institut de Sélection Animale) brown layers. We determined egg quality by the changes of physical characterization under storage conditions. The study used Restricted maximum likelihood and analysis of variance methods to assess the efficiency of fixed and mixed effect models. Results showed that the physical components of the egg were significantly affected at 5 ^*°*^*C*, 19.5 ^*°*^*C*, and 30 ^*°*^*C*(P<0.05). The effect was more adverse on eggs stored at 30 ^*°*^*C* for 32 days. However, storage temperatures of 5 ^*°*^*C* and 19.5 ^*°*^*C* led to an extensive reduction in the Haugh unit, yolk index, and egg white height. On the other hand, it increased the weight loss and albumen diameter under storage for 2^*nd*^, 12^*th*^, 22^*nd*^ and 32^*nd*^-time intervals. Based on these findings, the study recommends 5 ^*°*^*C* for egg quality preservation. The eggs should be refrigerated for 32 days, stored at 19.5 ^*°*^*C* for 14 days, and lastly kept at 30 ^*°*^*C* for a maximum of 7 days. The fixed-effect models exhibited more minor variances in diameter and height of albumen, yolk index, weight loss, and Haugh unit. This overlapped instances where the fixed-effect models were significantly the same as the mixed-effect models. This study proposes that the fixed effect model is the most appropriate for randomized completely block design experiments.

## 1 Introduction

Globally, there is a tremendous and ongoing human population increase over the past few years. Current geographical and statistical models predict 10 billion human beings approximately by around 2050 (Godfray et al., 2010; Kwasek, 2012; Popkin et al., 2012). The world population of humans was about 8 billion; this is considered over-grown as per 2017 (Mottet and Tempio, 2017). Rapid population growth in Africa contributes to at least half of the world's inhabitants growing from 1.1 billion to 2.4 billion (Alexandratos and Bruinsma., 2012; Latham, 2021). This growth is a result of the densely young generation population that is capable of reproduction. Such an increase implies an intense demand for basic human needs, which the world might find difficult to sustain (Hall et al., 2017). Food security and "food for the stomach" are immediate demands for human existence, such as shelter and clothing (Kwasek, 2012). Nevertheless, food security depends on the agricultural sector in several countries worldwide (Osabohien et al., 2018).

Poultry production is a broad area in animal keeping with an acknowledgeable contribution to secure food in several countries (Addo, 2017). Chicken eggs are a significant source of protein for human nutrition from poultry, widely preferred due to their relatively low price (Miller, 2019). Yet, chicken eggs are highly perishable food and may lose quality rapidly if not subjected to proper care from the time of laying, collection, to consumption (Molnár et al., 2016). Internal and external structures such as the Haugh unit, which measures freshness, describe egg quality (Lee et al., 2016). The shell does not cover the interior part impeccably and can be easily affected by the storage condition (Molnár et al., 2016). Joseph et al. (2018) observed a high rate of chicken egg consumption in Sub-Saharan Africa. The locally produced poultry products experience various challenges in meeting the current demand (Mottet and Tempio, 2017). That situation is not different in Kenya! As the population increases, the need for food also increases (Omondi, 2019).

Various stakeholders have been struggling to put in place measurements that could help maintain commercial egg quality from the time laid, collection to consumption. However, the quality has been inferior due to the extensive systems used (Manhique et al., 2017). For many farmers and retailers, egg handling and storage have subjected them to significant losses brought about by storage temperatures and storage duration. Egg quality for market value deteriorates with increased storage time and extreme storage temperatures (Lee et al., 2016). The storage temperature has a high impact on the yolk characteristics, shell thickness and increases airspace diameter. To a considerable extent, the egg white, a measure of protein composition, also deteriorates drastically (Osabohien et al., 2018)

However, the statistical analysis and interpretation of experiments based on commercial egg quality using randomized complete block design may be less critical if there is a failure to sufficiently understand the best model to be fitted. Since 1919, there is a higher rate of development in statistical techniques and their application to design and analyze agricultural experiments. A famous statistician Ronald A. Fisher performed statistical analysis of field and laboratory accumulated experiments (Yang, 2010). He invented Analysis of Variance (ANOVA) and different designs in experiments used since then in field and laboratory experiments to date. The invention was mainly to accurate estimators of the unknown parameter by eliminating bias and improving precision. Modelling standard randomized complete block design (RCBD) generates a mixed effect model (LeMay and Robinson, 2004).

Conversely, treating blocks as a fixed effect is usually the most appropriate choice (Dixon, 2016). Failure to differentiate between fixed versus random effects within an RCBD model has led to significant confusion and uncertainty with mixed model analysis (Yang, 2010). Discourse has risen as to the suitable way to state the hybrid linear model in experimental design (Bremer, 1993). According to Festing (2010), the inability to standardize the nomenclature of RCBD can confuse when teaching the principles of experimental design. Fitting this model gives a standard model, which is a mixed effect model. Therefore, the choice of factors as either fixed or random in a standard RCBD model has a significant consequence in statistical analysis;

(1) $$y_{ijk}=\mu +\lambda_{i}+\gamma_{j}+\varepsilon_{ijk}$$

whereμ is the grand mean, $\lambda_{i}$ is the $i^{th}$treatment effect, $\gamma_{j}$ is the $j^{th}$ block effect, and$\varepsilon_{ijk}$ residual error which is independent and associated with the observed value$y_{ijk}$. Whenever both $\lambda_{i},$$\gamma_{j}$ and are fixed, then the resulting model is fixed. On the other hand, if either$\lambda_{i}$ or $\gamma_{j}$is random and the other fixed, then the resulting model is mixed (Dixon, 2016). This paper investigates the effect of storage temperature at three levels (5 ^*°*^*C*, 19.5 ^*°*^*C*, 30 ^*°*^*C*) and storage duration at four levels (2^*nd*^, 12^*th*^, 22^*nd*^, and 32^*nd*^) on the egg's quality determinants using fixed and mixed-effect model.

## 2 Materials and methods

### 2.1 Egg collection and storage

According to Marzec et al. (2019), different egg storage conditions significantly affect both external and internal egg quality as hens age. This study targeted eggs from old ISA brown layers. We obtained a sample of 618 fresh and unfertilized eggs from 1,320 ISA brown layers at the University of Embu, Kirata, and Rana poultry farms using a simple random sampling technique without replacement. The exclusion criterion was used to disregard eggs laid by old ISA brown chicken the previous day and also eggs that had shapes other than average.

The weight of each of these fresh eggs was measured and marked. This study classified eggs into large (60–64) *g*, extra-large (65–69) *g,* and jumbo (70 and above) *g* sizes as described by (Altuntas and Sekeroglu, 2010). The eggs were then safely conveyed in carton trays to the Zoology research laboratory of the University of Embu. This study sampled 206 eggs at random from the 618 eggs. We then stored the eggs at 19.5 ^*°*^*C* room temperature. The remaining 412 eggs were randomly placed in plastic egg cartons 206 and stored under different storage temperature conditions (refrigerator 5 ^*°*^*C* and incubator 30 ^*°*^*C*). Other temperature conditions being treatments, and storage duration, acted as blocks.

### 2.2 Evaluation of egg quality

After storage, a sample of 52 eggs was randomly picked from every storage temperature condition at a time at a periodic interval of days; that is 2^*nd*^, 12^*th*^, 22^*nd*^, and 50 eggs on the 32^*nd*^ day. Each egg was picked and cracked over a flat surface to reduce the likelihood of shell shards breaking into the egg. The height of yolk and egg white were measured using a spherometer. The yolk, egg weight, and shell weight were measured by electronic sensitive digital weighing balance. We used Vanier callipers to measure the magnitude of yolk, shell, and white egg diameter. The average of the widest and the narrowest horizontal circumference was measured as the yolk diameter. Moreover, the standard of the broadest flat rim enclosed by the egg white was calculated as the albumen diameter as documented by Hegab and Hanafy (2019); Oleforuh-Okoleh and Eze (2016), and Sola-Ojo et al. (2016). Haugh unit (HU) was determined using the following procedure Haugh (1937) and Tran and Soottawat (2018);

$$HU=\;100\text{ ​}log_{10}\left(H_{h}-\;1.7\text{ ​}{W_{w}}^{0.37}+\;7.56\right)$$

where; HU=Haugh unit, $H_{h}=$ the height of the egg white (mm), and $W{\;}_{w}=$the egg's weight (g).

Weight loss was measured as the IEW−CEW.

IEW=The initial weight of the individual egg (g) and CEW=weight measured at the end of storage.

### 2.3 Data analysis

For model adequacy, we performed analytical tests on residual properties through the student zed residual method in SAS 9.4 for fixed and mixed effect models.

This study used Eq. (1) to formulate fixed-effect models associated with the interaction effect of the storage conditions (Wu et al., 2009);

(2) $$y_{ijk}=\mu +\lambda_{i}+\gamma_{j}+{\left(\lambda *\gamma \right)}_{ij}+\varepsilon_{ijk};\;i=1,\cdots ,w_{1},j=1,\cdots ,w_{2},k=1,\cdots ,w_{ij}$$

where μ= the grand mean, $\lambda_{i},$$\gamma_{j},$ ${\left(\lambda *\gamma \right)}_{ij}$and $\varepsilon_{ijk}$are independent random variables and follow a normal distribution with variance$\sigma_{\lambda }^{2}$$\sigma_{\gamma }^{2}$$\sigma_{\lambda \gamma }^{2}$. The means of these variables are all zero (Khuri, 2000). The matrix notation of the model in Eq. (2) was further expressed as

(3) $$y=A\mu +A_{1}\lambda +A_{2}\gamma +A_{3}\left(\lambda *\gamma \right)+\varepsilon$$

Using Eq. (3), we supposed that λ∗γ = ψ, which is the effect of interaction between λ andγ, this study had that;

(4) $$y=A\mu +A_{1}\lambda +A_{2}\gamma +A_{3}\left(\psi \right)+\varepsilon$$

where $A=I_{\sum_{i}\sum_{j}w_{ij}}$. Further, given that$\varepsilon_{ijk}$ is a random error whose entries are in respect to observed values y, ε~$N\left(0_{\sum_{i}\sum_{j}w_{ij}},\sigma_{\sigma_{\sum_{i}\sum_{j}w_{ij}}}^{2}\right)$, (Stroup and Littell, 2002). We used Eq. (4) to formulate the response variables for the experiment.

### 2.4 Determination model of efficiency of the models

We determined the efficiencies of the fixed and mixed-effect models through estimation of variance components using the method of ANOVA and restricted maximum likelihood (REML).

#### 2.4.1 Analysis of variance method

According to Corbeil and Searle (1976), analysis of variance procedure is considered for the fixed effect model. We formulated the structure of the linear Equation depended on RCBD. Letting $V_{i}^{2},i=1,2,\cdots ,r+1$ denote the second-order moment error for the $i^{th}$ variation source in the model (Equation 4). We expressed $V_{i}^{2}$ as; $V_{i}^{2}=y^{T}R_{i}y$; $R_{i}\in {ℜ}^{r}$ in such a way that; $X^{T}R_{i}X=0_{rr}$ where $X^{T}R_{i}$ is an orthogonal vector to all columns of X and thus,

(5) $$E\left(V_{i}^{2}\right)=tr\left(R_{i}\Sigma \right)+X\beta^{T}R_{i}\left(X\beta \right)=tr\left(\sum_{q=1}^{r+1}\psi_{q}R_{i}A_{q}\right)=\sum_{q=1}^{r+1}\psi_{q}tr\left(X_{q}^{T}R_{i}X_{q}\right)$$

$V_{i}^{2}$ in Eq. (5) depends only on the estimated variance component (Heba et al., 2015).

But, $V=\left[\begin{array}{l} S_{1}^{2}\\ \vdots \\ S_{r+1}^{2} \end{array}\right]\;\text{and}\;\psi =\left[\begin{array}{l} \psi_{1}\\ \vdots \\ \psi_{r+1} \end{array}\right]E\left(S\right)=Z\psi ,Z=\left[\begin{array}{lll} tr\left(X_{1}^{T}R_{1}X_{1}\right) & \cdots & tr\left(X_{r+1}^{T}R_{1}X_{r+1}\right)\\ \vdots & \ddots & \vdots \\ tr\left(X_{1}^{T}R_{r+1}X_{1}\right) & \cdots & tr\left(X_{r+1}^{T}R_{r+1}X_{r+1}\right) \end{array}\right]$.

#### 2.4.2 Restricted maximum likelihood procedure (REML)

This method is a more general method majorly used to estimate a bias-free variance component in a mixed effect model. This study expressed Eq. (4) further as; y=Xβ+Zb+ε, Where, $y=\left[\begin{array}{l} y_{1}\\ \vdots \\ y_{w} \end{array}\right]$, $X=\left[\begin{array}{l} X_{1}\\ \vdots \\ X_{w} \end{array}\right]$, $Z=\left[\begin{array}{lll} Z_{1} & \cdots & 0\\ \vdots & \ddots & \vdots \\ 0 & \cdots & Z_{w} \end{array}\right]$, $b=\left[\begin{array}{l} b_{1}\\ \vdots \\ b_{w} \end{array}\right]$. In this case ε~N(0,u(θ)) with $u\left(\theta \right)=\left[\begin{array}{lll} u_{1}\left(\theta \right) & \cdots & 0\\ \vdots & \ddots & \vdots \\ 0 & \cdots & u_{w}\left(\theta \right) \end{array}\right]$ general covariance matrix parametrized by θ. If (2) is true, then $y^{T}R_{i}y$is the contrast error. It is only possible to come out with at most w−rsuch vectors which are linearly independent. It follows that $X^{T}R_{i}X=0_{rr}$ and $\left\{X^{T}R_{i}y\right\}=0_{rr}$. This can be expressed further as $A^{T}y=0$, where $X^{T}R_{i}=A^{T}$. We defined a contrast error vector by $c=A^{T}y\left(X\beta +Zb+\varepsilon \right)=A^{T}\varepsilon \sim N\left(0,A^{T}u\left(\theta \right)\right)$ which does not contain any element of unknown parameter β and b. The absence of these unknown parameters gives sufficient information about θ when inferred based on c rather than y(Zhang, 2015). Therefore;

(6) Lc=(θ|ATy)=−12logdetu−12logdetXTu−1X−12(y−Xβˆ)Tu−1(y−Xβˆ)−12logdet∏i=1rui−12logdet∏i=1rXiTui−1Xi−12∑1=1r(yi−Xiβˆ)Tui−1(yi−Xiβˆ)−12∑1=1rlogdetui−12∑i=1rlogdetXiTui−1Xi−12∑1=1r(yi−Xiβˆ)Tui−1(yi−Xiβˆ)

We then maximized $L_{c}=\left(\theta |A^{T}y\right)$in Eq. (6) for $u_{i}$. The detailed derivation is found in (Lindstrom and Bates, 1988). Lastly, we tested the significant difference of the variance component by the least significant difference.

## 3 Results and discussion

### 3.1 Effects of storage conditions on both exterior and interior components of the egg

#### 3.1.1 Egg weight loss

Table 1 displays the effect of different storage durations on egg quality for large (60–64) *g*, extra-large (65–69) *g* and jumbo (70 and above) *g* sizes. R-Square (R-Sq.) of the egg weight loss (EWL) indicates that the fixed model was 93%, 85% and 83% for the egg sizes. The models were good at explaining the total egg weight loss during the storage duration. The large (60–64) *g* egg size highly explained variation*.* The coefficient of variation of EWL was below 30%, hence accurate measurements. The jumbo (70 and above) *g* had the highest coefficient of variation (18%). The effect of storage time on the egg weight was remarkable at 95% confidence intervals with the least significant difference (LSD) 0.34, 0.34 and 0.39, respectively (Table 1). The mean weight losses were 1.67 *g*, 1.78 *g*, and 1.99 *g,* with jumbo (70 and above) *g* recording the highest loss. Table 3 presents the effects of the interaction of storage temperature and duration within the fixed-effect models for every category of egg size.

**Table 1:** Effect of storage time as a fixed effect in the fixed-effect model.

Egg size	Period	EWL	SW	SD	AH	AD	HU	YI
Large (60–64) *g*.	2nd	0.57	7.53	0.085	6.6	6.77	79.51	0.3507
	12th	1.11	7.42	0.077	4.06	7.85	56.81	0.3258
	22nd	2.01	7.38	0.073	3.51	8.03	49.82	0.2867
	32nd	3.01	7.17	0.062	1.84	8.85	17.02	0.2296
	Mean	1.67	7.37	0.074	4	7.87	50.79	0.2982
	CV	16.97	8.52	14.53	11.66	13.07	14.14	6.117
	P-Value	0.0002	0.0475	0.0215	0.0195	0.0018	0.0139	0.0002
	LSD	0.340	0.6775	0.0041	1.102	1.050	5.074	0.186
	R-Sq.	0.93	0.69	0.68	0.73	0.62	0.84	0.85
Extra-large (65–69) *g.*	2nd	0.59	8.75	0.0845	6.45	7.88	76.83	0.3409
	12th	1.13	8.64	0.0765	4	9.07	53.46	0.3266
	22nd	2.33	8.61	0.0725	2.65	9.64	31.62	0.278
	32nd	3.07	8.39	0.0619	1.79	10.25	8.32	0.2115
	Mean	1.78	8.59	0.0738	3.72	9.312	42.56	0.2893
	CV	17.34	8.895	14.9	9.95	11.36	12.43	4.41
	P-Value	0.0001	0.0382	0.0112	0.0202	0.002	0.0141	0.0004
	LSD	0.3408	0.6781	0.0047	1.1029	1.0514	5.0753	0.1866
	R-Sq.	0.8542	0.6174	0.7034	0.6699	0.6185	0.7819	0.829
Jumbo (70 and more) *g*.	2nd	0.62	9.23	0.084	6.51	7.96	76.07	0.3325
	12th	1.21	9.12	0.077	4.07	9.38	52.15	0.2865
	22nd	2.75	9.08	0.072	2.62	10.26	27.26	0.2389
	32nd	3.36	8.87	0.061	1.78	12.01	3.99	0.1669
	Mean	1.99	9.079	0.074	3.75	9.9025	39.87	0.2562
	CV	18.14	9.695	15.7	11.48	12.89	13.96	5.933
	P-Value	0.0009	0.0561	0.0522	0.0202	0.0025	0.0146	0.0009
	LSD	0.3918	0.7791	0.0057	1.1039	1.0524	5.0763	0.1876
	R-Sq.	0.8322	0.5954	0.814	0.8619	0.6805	0.7739	0.92

The interaction effect of egg weight loss indicates higher losses on the 32^*nd*^ day of storage in 5 ^*°*^*C*, 19.5 ^*°*^*C* and 30 ^*°*^*C* for all the egg sizes. The diluents of the egg content from side to side of the shell, probably due to evaporation led to weight loss. These findings are in agreement with Fasenko et al. (2001); Hassan et al. (2005); Reijrink et al. (2010); Alsobayel and Albadry (2011); Akter et al. (2014) and Yimenu et al. (2011), who attested that total egg weight reduces with prolonged storage duration. This study realized insignificant weight loss of extra-large (65–69) *g* egg size stored at 5 ^*°*^*C*. However, jumbo (70 and above) *g* had a significant difference on the 32^*nd*^ day.

Table 2 presents the effect of storage conditions on the physical characterization of the egg with temperature as a fixed effect. The weight loss increased from low (5 ^*°*^*C*) to high (30 ^*°*^*C*) in large (60–64) *g*, extra-large (65–69) *g* and jumbo (70 and above) *g* sizes. The trend of weight loss was similar in the mixed-effect model. However, weight loss increased as the egg size increases. The effect was highly significant in general *P* < 0.05 with LSD of 0.352, 0.344 and 0.35, respectively, as shown in Table 2. The temperatures under storage influence a reduction in egg weight. Consequently, eggs subjected to low-level temperature 5 ^*°*^*C*; exhibited a lesser reduction in egg content than room temperature. Loss of water and other gaseous components was slight on eggs stored under low-level temperature 5 ^*°*^*C* compared to 19.5 ^*°*^*C*. These outcomes support Samli et al. (2005) and Hasan and Alylin (2014), who observed a decline in the egg's weight at 29 ^*°*^*C* with the 10^*th*^ day of storage.

**Table 2:** Effect of temperature as fixed effect in the fixed-effect model.

Egg size	Temp.	EWL	SW	SD	AH	AD	HU	YI
Large (60–64) *g.*	room (19.5*°C*)	1.77	7.31	0.071	3.28	7.93	51.85	0.2802
	Ref. (5*°C*)	0.85	7.72	0.091	6.11	7.16	70.67	0.357
	Inc. (30*°C*)	2.37	7.09	0.059	2.66	8.56	29.84	0.2583
	P-Value	0.0006	0.0018	0.0014	0.0014	0.0086	0.0031	0.0002
	LSD	0.352	0.558	0.0315	0.926	0.881	8.696	0.132
	Var. Estimate	0.1393	0.3792	0.0391	1.108	0.706	67.47	0.0107
Extra-large (65–69) *g*.	room (19.5*°C*)	1.84	8.67	0.066	3.02	9.38	40.79	0.275
	Ref. (5*°C*)	0.86	9.75	0.091	5.89	7.7	63.95	0.3546
	Inc. (30*°C*)	2.64	7.36	0.062	2.25	10.87	22.93	0.2314
	P-Value	0.00083	2.50E-05	0.0016	0.0015	0.0088	0.0033	0.0004
	LSD	0.3435	0.5491	0.02231	0.9169	0.8723	8.6872	0.1234
	Var. Estimate	0.1306	0.3704	0.0303	1.099	0.6972	67.46	0.002
Jumbo (70 and more) *g.*	room (19.5*°C*)	1.951	8.57	0.064	3.21	9.01	38.48	0.261
	Ref. (5*°C*)	0.918	12.18	0.099	5.89	7.98	63.17	0.341
	Inc. (30*°C*)	3.087	6.46	0.057	2.15	12.73	17.95	0.166
	P-Value	0.0009	0.0001	0.0017	0.0017	0.0089	0.0034	0.0005
	LSD	0.35	0.56	0.031	0.93	0.88	8.703	0.1396
	Var. Estimate	0.1293	0.3692	0.02911	1.098	0.696	67.46	0.0007

##### 3.1.1.1 Eggshell weight

Table 1 also illustrates the total variation in storage time and temperature in the fixed RCBD model of the shell weight (SW). The results showed that R-sq. of 69%, 62% and 67% for large (60–64) *g*, extra-large (65–69) *g*, and jumbo (70 and above) *g* sizes, respectively. These models explained over 50% of the SW variation. The slightest variation of the three categories of egg sizes was explained by extra-large (65–69) *g*. The data collected on SW was good at 90%, as defined by the coefficient of variation <10% for the egg sizes. The SW model was significant for large (60–64) *g P* = 0475 and extra-large (65–69) *g P* = 0.0382 The finding on significant levels was in line with Samli et al. (2005), who reported a substantial effect of storage duration and storage temperature on shell weight. Conversely, the SW model was not significant for jumbo (70 and above) *g* with *P* > 0.05 Thus, the SW of jumbo (70 and above) *g* size was not affected by the storage duration.

We observed the LSDs as 0.6944, 0.6781 and 0.7791, with the highest value in jumbo (70 and above) g size. Even though the SW model was insignificant in jumbo (70 and above) g, this study realized the highest great SW of 9.079 *g*. The interaction effect on SW in different storage duration was significantly different from 2^*nd*^, 12^*th*^, 22^*nd*^, and 22^*nd*^ *P* > 0.05 in large (60–64) *g*. It was very different on the 32^*nd*^ *P* < 0.05 The insignificant effect of the storage for this study was also in line with Akter et al. (2014), who reported no relationship between shell weight, temperature, and storage time.

Similarly, the effect of different storage temperatures was not significantly different from each other at 5 ^*°*^*C* and 19.5 ^*°*^*C* temperatures of storage, as shown in Table 3. However, we realized a significant difference SW for the extra-large (65–69) *g* and jumbo (70 and above) *g* sizes *P* < 0.05. There was a substantial difference at 30 ^*°*^*C*. So, egg retailers could store eggs at room temperatures or 5 ^*°*^*C* for 22 days with maintained SW. There was a significant difference in SW in the mixed effect model between 19.5 ^*°*^*C* and 30 ^*°*^*C*. Measurements of SW in 30 ^*°*^*C* and 5 ^*°*^*C* also differed significantly *P* < 0.05. In contrast, the difference between 5 ^*°*^*C* and 19.5 ^*°*^*C* was infinitesimal.

**Table 3:** Effect of interaction effect in the fixed-effect model.

Egg Size	Temp.	Period (day)	EWL (*g*)	SW (*g*)	SD (*cm*)	AH (*mm*)	AD (*cm*)	HU	YI
Extra-large (65–69) *g*	19.5*°C*	2nd	0.34^a^	8.88^a^	0.11^a^	8.38^a^	7.34^a^	77.84^a^	0.32^a^
		12th	0.91^a^	7.96^a^	0.099^a^	7.61^a^	8.04^a^	67.91^b^	0.308^a^
		22nd	2.12^b^	7.63^a^	0.061^a^	6.12^b^	9.58^b^	63.03^c^	0.27^b^
		32nd	3.02^c^	6.95^c^	0.02^b^	5.22^c^	12.27^c^	56.27^bc^	0.23^b^
	5*°C*	2nd	0.11^a^	8.91^a^	0.13^a^	8.65^a^	7.27^a^	83.63^d^	0.35^a^
		12th	0.43^a^	8.41^a^	0.12^a^	7.85^a^	7.65^a^	80.23^d^	0.34^a^
		22nd	0.76^a^	8.01^a^	0.11^a^	7.55^a^	8.19^a^	76.28^a^	0.32^a^
		32nd	1.11^a^	7.91^a^	0.04^b^	7.45^a^	9.11^b^	64.15^c^	0.25^b^
	30*°C*	2nd	1^a^	8.34^a^	0.09^a^	7.45^a^	7.42^a^	72.5^a^	0.27^b^
		12th	1.11^a^	7.42^a^	0.054^a^	6.01^b^	8.88^a^	62.33^c^	0.263^b^
		22nd	2.33^b^	6.49^c^	0.03^b^	5.75^b^	10.73^b^	53.68^bc^	0.24^b^
		32nd	3.89^d^	4.57^d^	0.01^b^	3.62^d^	12.73^c^	31.43^abd^	0.2^c^
Extra-large (65–69) *g*	19.5*°C*	2nd	0.38^a^	8.89^a^	0.11^a^	8.24^a^	7.36^a^	71.76^a^	0.31^a^
		12th	1.01^b^	7.63^a^	0.098^a^	6.47^c^	8.12^a^	65.83^b^	0.298^a^
		22nd	2.35^ab^	6.21^b^	0.06^b^	5.98^b^	9.66^b^	64.95^b^	0.26^b^
		32nd	3.09^c^	5.95^b^	0.02^b^	5.08^b^	12.35^ab^	54.19^c^	0.22^bc^
	5*°C*	2nd	0.14^a^	9.91^ab^	0.13^a^	8.52^a^	7.29^a^	84.55^d^	0.35^d^
		12th	0.77^a^	9.47^ab^	0.12^a^	7.71^a^	7.73^a^	81.15^d^	0.33^a^
		22nd	0.97^b^	8.81^a^	0.11^a^	7.42^ab^	8.27^a^	77.2^a^	0.31^a^
		32nd	1.81^ab^	7.93^a^	0.04^b^	7.32^ab^	9.19^b^	64.07^b^	0.24^b^
	30*°C*	2nd	1.13^b^	8.34^a^	0.06^b^	8.01^a^	7.44^a^	70.42^a^	0.26^b^
		12th	1.7^ab^	7.22^a^	0.053^b^	6.87^c^	8.96^a^	60.25^b^	0.253^b^
		22nd	3.03^c^	6.62^b^	0.03^b^	5.61^b^	10.81^b^	52.6^c^	0.23^b^
		32nd	4.1^ac^	4.44^c^	0.01^c^	3.48^c^	12.81^ab^	29.35^f^	0.2^c^
Jumbo (70 and more) *g*	19.5*°C*	2nd	0.5^a^	10.85^a^	0.11^a^	8.02^a^	7.47^a^	70.55^a^	0.29^a^
		12th	1^a^	7.63^b^	0.09^a^	7.25^c^	8.14^a^	55.62^b^	0.28^a^
		22nd	2.7^b^	6.21^b^	0.06^a^	5.76^b^	9.68^b^	54.74^b^	0.24^b^
		32nd	3.1^ab^	5.95^b^	0.02^b^	4.86^b^	12.37^c^	43.98^ab^	0.2^c^
	5*°C*	2nd	0.16^a^	11.47^a^	0.13^c^	8.99^a^	7.37^a^	85.34^c^	0.33^ab^
		12th	0.83^a^	10.56^a^	0.12^c^	7.49^c^	7.75^a^	79.94^c^	0.31^a^
		22nd	1.07^a^	9.38^a^	0.11^a^	7.19^c^	8.29^a^	71.99^a^	0.29^a^
		32nd	1.31^b^	6.93^b^	0.04^b^	7.09^c^	9.21^b^	60.86^b^	0.22^b^
	30*°C*	2nd	1.19^a^	10.22^a^	0.06^a^	8.09^a^	7.52^a^	68.21^a^	0.24^b^
		12th	1.9^b^	8.1^b^	0.05^a^	6.65^c^	8.98^a^	50.04^b^	0.24^b^
		22nd	2.73^b^	4.62^c^	0.03^b^	5.11^b^	10.83^d^	39.39^d^	0.21^c^
		32nd	4.4^c^	3.4^d^	0.01^b^	3.26^d^	12.83^f^	19.14^f^	0.17^d^

Different letters denote significant difference (P < 0.05) within different categories of egg size.

From Table 2, SW reduced from low (5 ^*°*^*C*) to high (30 ^*°*^*C*) in large (60–64) *g*, extra-large (65–69) *g* and jumbo (70 and above) *g* sizes as in the mixed-effect model. This study detected an increase in reduction in SW as the egg size increases. The inter-temperature decrease of SW increases large (60–64) *g* to jumbo (70 and above) *g* sizes. This indicated that the rate of water loss and other vaporous components was higher in larger sizes of eggs. The effect was significant *P* < 0.05 with LSD of 0.558, 0.549, and 0.369, respectively, as shown in Table 2. Hence, temperature significantly affected the SW.

##### 3.1.1.2 Eggshell diameter

The R-Sq. of the shell diameter (SD) showed that the fixed model was 68%, 70% and 81% for the large (60–64) *g*, extra-large (65–69) *g* and jumbo (70 and above) *g* egg sizes, respectively as shown in Table 1. The fixed-effect models were statistically good at explaining the variation during the storage. The variation in shell diameter was highly explained in the jumbo (70 and above) *g* while lower in large (60–64) *g*. The coefficient of variation of SD was below 30%, which showed that the study data was accurate. The study found that jumbo (70 and above) *g* had the highest coefficient of variation (16%). The effect of storage time on the SD was notable at a 5% level of significance with LSD 0.0041, 0.0047 and 0.0061, respectively, as shown in Table 1. It was insignificant in jumbo (70 and above) *g P* > 0.05 Which pointed out that shell thickness was not necessarily affected by the storage duration. The mean SD was 0.074 cm, 0.0738 cm and 0.074 cm with jumbo (70 and above) *g*, which recorded the highest thickness value. From the results in Table 1 and Table 2, shell thickness reduces with an increase in egg size.

This finding of shell thickness agrees with Altuntaş & Şekeroǧ;lu (2008) who argued that the strength required to initiate rapture on the z-axis declines with an increase in egg size. This weakening caused a reduction of shell thickness from the large (60–64) *g*, extra-large (65–69) *g* and jumbo (70 and above) *g* egg sizes. Table 2 shows the effect of storage temperature on SD. The SD reduced from low (5 ^*°*^*C*) to high (30 ^*°*^*C*) in large (60–64) *g*, extra-large (65–69) *g* and jumbo (70 and above) *g* sizes similar to the mixed-effect model. The study detected an increase in reduction in SD with an increase in egg size. The inter-temperature decrease of SD increases large (60–64) *g* to jumbo (70 and above) *g* sizes revealing that the rate of water loss and other vaporous components was higher in larger sizes of eggs. The effect was significant *P* < 0.05 with LSD of 0.558, 0.549 and 0.369, respectively, as shown in Table 2, signifying that temperature expressively affected the SD. The finding in the mixed effect model contradicts Saleh et al. (2020) and Akter et al. (2014), who found an insignificant effect on SD. However, this study observed a significant interaction effect in the fixed-effect model.

##### 3.1.1.3 Egg albumen height

Table 1 also illustrates the total variation explained by the conditions in the fixed RCBD model of the albumen height (AH). The R-Sq. of 73%, 67%, and 86% for large (60–64) g, extra-large (65–69) *g*, and jumbo (70 and above) *g* sizes, respectively. The fixed-effect model explained over 50% of the AH variation. The jumbo (70 and above) *g* egg size model explained the highest variation. The measurements on AH were good at 85%, as defined by the coefficient of variation <15% for the egg sizes. Storage duration significantly affected the albumen height for all the egg sizes *P* < 0.05 The LSD was observed as 1.102, 1.103 and 1.103. These LSD values were almost the same as the three egg size classes.

The effect of storage temperature on AH exists in Table 2. The AH reduced from low (5 ^*°*^*C*) to high (30 ^*°*^*C*) in large (60–64) *g*, extra-large (65–69) *g* and jumbo (70 and above) *g* sizes, similar to the mixed-effect model. We noticed a general increase in the reduction of AH as the egg size increases. The effect was significant *P* < 0.05 with LSD of 0.926, 0.917, and 0.88, respectively, as shown in Table 2, indicating that temperature significantly affected the AH. Albumen height decreased from 6.6 mm to 1.84 mm*.* The effect of AH on the 2^*nd*^ and 12^*th*^ days was remarkably the same, recording the highest peak. It was different from 22^*nd*^ and 32^*nd*^ (*P* < 0.05) The impact at 22^*nd*^ and 32^*nd*^ periods were significantly different from those at 30 ^*°*^*C* and 32^*nd*^*.* Jumbo (70 and above) *g* sizes recorded the smallest AH. Further, this study noticed no difference in AH at 5 ^*°*^*C* and 19.5 ^*°*^*C* (*P* > 0.05) However, there was a significant difference in AH at 30 ^*°*^*C* (*P* < 0.05) for all the egg sizes. The eggs stored at 5 ^*°*^*C* recorded the highest AH on the 2^*nd*^ day of storage, as shown in Table 3.

##### 3.1.1.4 Egg albumen diameter

The R-Sq. of the albumen diameter (AD) pointed out that the fixed models were 62%, 61% and 68% for the egg sizes. The fixed-effect models were statistically good in explaining the variation of the AD during the storage. The jumbo (70 and above) *g* explained the highest value (68%), implying that most variation due to storage conditions was experienced by jumbo (70 and above) *g*. The coefficient of variation of the AD fixed-effect model was below 15%, which showed that the study was precise. They established that jumbo (70 and above) *g* had the highest coefficient of variation (13%) while extra-large (65–69) *g* had the lowest. The effect of time on the AD was highly significant at 95% confidence intervals with LSD, 1.05, as shown in Table 1. The means of AD were 7.87 cm, 9.31 cm, 9.9 cm*,* with jumbo (70 and above) *g* recording the highest thickness value, inferring that the horizontal circumference covered by the albumen generally increased with the egg size. Therefore, a small egg size covered a smaller circumference. From the results in Table 1 and Table 2, AD rises with an increase in egg size.

Our finding agrees with Altuntaş & Şekeroǧ;lu (2008), who argues that the egg internal components increase with egg size. Given that a smaller egg size smaller circumference, albumen diameter increased from the large (60–64) *g*, extra-large (65–69) *g* and jumbo (70 and above) *g* egg sizes. Table 2 presents the effect of storage temperature on AD. The AD increases from low (5 ^*°*^*C*) to high (30 ^*°*^*C*) in large (60–64) *g*, extra-large (65–69) *g,* and jumbo (70 and above) *g* sizes, similar to the mixed-effect model. We witnessed an increase in AD as the egg size increases. The inter-temperature decrease of AD increases large (60–64) *g* to jumbo (70 and above) *g* sizes. The rate of water loss and other vaporous components was higher in larger sizes of eggs. The effect was significant (*P* < 0.05) with LSD of 0.881, 0.8723, and 0.88 respectively, as shown in Table 2, demonstrating that temperature significantly affected AD. The interaction effect on HU (large (60–64) *g*) was insignificant at 5 ^*°*^*C*, 19.5 ^*°*^*C,* 30 ^*°*^*C* for the 22^*nd*^ and 12^*th*^ time.

##### 3.1.1.5 Haugh Unit

The effect of different storage periods on Haugh Unit (HU) for large (60–64) *g*, extra-large (65–69) *g*, and jumbo (70 and above) *g* sizes was expressed in Table 1. The R-Sq. of the HU shows that the fixed model was 85%, 78%, and 77% for the egg sizes, respectively. These models explained 70% of the effect caused by storage conditions. The large (60–64) *g* expressed the highest variation while the lowest in jumbo (70 and above) *g* sizes had the lowest. The coefficient of variation of HU was below 15%, which shows that the measurements were keenly observed. The large (60–64) *g* had the highest coefficient of variation (14%). The effect of storage time on the HU was significant at 95% confidence intervals with (LSD) of 0.19. The confidence interval was approximately similar for the three classes of egg sizes, as shown in Table 1. The means of HU were 50.79, 42.56, 39.87, with jumbo (70 and above) *g* recording the lowest value. HU reduced with an increase in the egg size. The observed HU of jumbo on the 12^*th*^ and 22^*nd*^ days were different (*P* < 0.05) at 5 ^*°*^*C*, 19.5 ^*°*^*C*, 30 ^*°*^*C*. A significant difference was observed on eggs stored for 32 and 2 days (*P* < 0.05) The effect of 2^*nd*^-day storage was significantly different from the 12^*th*^ and 22^*nd*^ days (*P* < 0.05) The impact of the 32^*nd*^ period was also quite different from the 12^*th*^ and 22^*nd*^. The HU reduced steadily from the 2^*nd*^ to 32^*nd*^ storage period.

Further, we observed an insignificant difference between the effect of 5 ^*°*^*C* and 19.5 ^*°*^*C* on the HU. The effect of 30 ^*°*^*C* was significantly different at 5 ^*°*^*C* and 19.5 ^*o*^ *C*. We also detected that HU at 5 ^*°*^*C* remained remarkably the same throughout the storage period used for this study.

From the results in Table 1 and Table 2, HU decreases with egg size. Our finding contradicts Emsley et al. (1977), who established that HU increases with egg size. Nevertheless, Kinney et al. (1970), Van Tijen and Kuit (1970), and Iposu et al. (1994) conformed with the current study. Table 2 shows the effect of storage temperature on HU. The HU declines from low (5 ^*°*^*C*) to high (30 ^*°*^*C*) in large (60–64) *g*, extra-large (65–69) *g* and jumbo (70 and above) *g* sizes. It signifies that eggs with more significant weight recorded lower HU, similar to the mixed-effect model. An increase in HU as the egg size increases. The inter-temperature decrease of HU increases large (60–64) *g* to jumbo (70 and above) *g* sizes. It demonstrates that the rate of loss of HU was higher in the jumbo (70 and above) *g* egg size. The rate of water loss and other vaporous components was more elevated in larger sizes of eggs. The effect was significant (*P* < 0.05) with LSD of 8.696, 8.687, and 8.703, respectively, as shown in Table 2. Thus, indicating that temperature significantly affected the HU.

#### 3.1.2 Yolk index

Table 1 also displays the total variation of the storage conditions in the fixed RCBD model of the yolk index (YI). The R-Sq. of 85%, 83%, 92% for large (60–64) *g*, extra-large (65–69) *g* and jumbo (70 and above) *g* sizes, respectively. The fixed-effect model explains over 50% of the SW variation. The slightest variation of the three categories of egg sizes was explained by extra-large (65–69) *g*. The obtained values on YI was 93%, as described by the coefficient of variation <7% for the egg sizes. The YI model with storage time was highly significant for the three eggs *P* < 0.05 Which implied that YI was affected by the storage duration. The weighty finding of the effect of storage duration is in line with Yimenu et al. (2011), who realized a decrease in YI as storage duration increases.

The LSDs observed were 0.186, 0.187, 0.188, with the highest value in jumbo (70 and above) *g* size. The significant differences between YI data points increased with an increase in egg size. The mean values of YI were 0.298, 0.289, 0.256, with large (60–64) *g* size recording the highest. Thus, the yolk index decreases with an increase in egg size.

From the results in Table 1 and Table 2, YI reduced with an increase in egg size. Table 2 shows the effect of storage temperature on YI. This study observed a related change of YI in both fixed and mixed-effect models. The YI reduces from low (5 ^*°*^*C*) to high (30 ^*°*^*C*) in large (60–64) *g*, extra-large (65–69) *g* and jumbo (70 and above) *g* sizes. The study observed a decrease in YI as the egg size increases. The inter-temperature reduction of YI increased from large (60–64) *g* to jumbo (70 and above) *g* sizes. The effect was significant *P* < 0.05 with LSD of 0.132, 0.1234 and 0.1396, respectively, as shown in Table 2. Validating that temperature significantly affected the YI.

### 3.2 Comparison of the model efficiency

In comparing the model efficiencies, the estimated variance components for the fixed-effect model (EWL) were 0.1393, 0.1306 and 0.1293 in Table 2. Each of these values was less than 0.2325, 0.2213, 0.2284 of the mixed-effect model in Table 4. Variance components of the mixed effect models (EWL) were significantly different from fixed-effect models *P* < 0.05 Further, the estimated variance components for the fixed-effect models (SW) were 0.379, 0.37 and 0.36, while those of the mixed-effect models were 0.3075, 0.3713 and 0.3578 in Table 4. Each of these values corresponding to the large (60–64) g, extra-large (65–69) *g* and jumbo (70 and above) *g* egg sizes was insignificantly different *P* > 0.05 This was in exception of large (60–64) *g* size, which had a significant difference *P* < 0.05 The mixed-effect and fixed-effect models were significantly the same P>0.05.

**Table 4:** The effect of temperature as fixed effect and storage duration as a random effect in the mixed-effect model.

	Temp.	EWL	SW	SD	AH	AD	HU	YI
Large (60–64) *g*.	room (19.5*°C*)	1.88	8.21	0.1	4.69	9.37	63.68	0.2717
	Ref. (5*°C*)	0.67	8.8	0.12	7.27	8.17	83.96	0.3631
	Inc. (30*°C*)	3.55	7.6	0.1	2.57	9.57	37.15	0.2497
	R.E	1.01	0.094	0.00005	0.31	0.054	32.7	0.0287
	Var. Est.	0.2325	0.3075	0.00048	1.707	0.7424	69.1302	0.0542
	P-Value	0.0019	0.0548	0.00912	0.0063	0.0112	0.0167	0.0004
Extra-large (65–69) *g*.	room (19.5*°C*)	1.908	8.2	0.1	4.69	9.36	61.35	0.2576
	Ref. (5*°C*)	0.698	8.79	0.12	7.27	8.16	82.52	0.3553
	Inc. (30*°C*)	3.578	7.59	0.079	2.57	9.56	29.83	0.2466
	R. E	1.02322	0.104	0.0012	0.321	0.064	32.7	0.03
	Var. Est.	0.2213	0.3713	0.00011	1.90744	0.94214	69.415	0.04394
	P-Value	0.00199	0.05489	0.00919	0.00635	0.01125	0.01675	0.00045
Jumbo (70 and more) *g.*	Room (19.5*°C*)	1.92	8.19	0.097	4.685	9.35	58.96	0.255
	Ref. (5*°C*)	0.71	8.78	0.122	7.265	8.15	81.07	0.348
	Inc. (30*°C*)	3.59	7.58	0.076	2.565	9.55	24.74	0.239
	Random effect	1.016	0.103	0.0012	0.321	0.064	32.7	0.0301
	Var. Estimate	0.2284	0.3578	0.0008	1.908	0.992	68.38	0.0445
	P-Value	0.0018	0.0547	0.009	0.0062	0.0111	0.016	0.0003

The fixed-effect models (AH) had 1.11, 1.099 and 1.098 estimated variance components, which were significantly lesser than those of the mixed-effect models; 1.707, 1.907 and 1.908 in Table 4, (*P* < 0.05) Moreover, the estimated variance components for the fixed-effect models (AD) were 0.706, 0.697 and 0.696. These values were significantly different from those of the mixed-effect model; 0.9424, 0.9421 and 0.9922 in Table 4. Consequently, the mixed-effect and fixed-effect model was not the same *P* < 0.05 Fixed effect model posted a small estimated variance component.

Furthermore, the estimated variance components for the fixed-effect models (HU) were 67.47, 67.46 and 67.46. Each of these values was significantly different from those mixed-effect models; 69.13, 69.41 and 68.38 in Table 4 (*P* < 0.05). Therefore, mixed and fixed-effect models was not the same. Fixed effect model posted small estimated variance component. Similarly, the estimated variance components for the fixed-effect models (YI) were 0.0107, 0.002 and 0.0007. These values were significantly different from those of the mixed-effect models; 0.0542, 0.0439 and 0.0445 in Table 4 *P* < 0.05 Hence mixed and fixed-effect models were not the same. Generally, fixed-effect models resulted in smaller estimated variance components hence more efficient. The models were significantly the same in shell thickness *P* > 0.05.

The results above clearly showed that the fixed-effect model exhibited a minor variance in yolk index, egg weight loss, Haugh unit, albumen diameter and albumen height. This overlapped instance where the fixed effect model was significantly the same as the mixed-effect model. Therefore, this study proposes that treating blocks as a fixed-effect in the RCBD experiment is appropriate. This finding was in line with a survey carried out by Dixon (2016), who suggested that the blocking effect in RCBD should be treated as a fixed effect. Conversely, our study disputes recommendations by LeMay and Robinson (2004) and Festing (2010), who stressed that RCBD experiments should be analysed as a mixed effect model.

### 3.3 Conclusion and recommendation

This study established that the determining factors of egg quality, physical components of the egg, were significantly different when we kept eggs at 5 ^*°*^C, 19.5 ^*°*^*C* and 30 ^*°*^*C*. The effect was more adverse on eggs stored at 30 ^*o*^ *C* for 32 days. Besides, storage temperatures of 5 ^*°*^*C* and 19.5 ^*°*^*C* led to a considerable reduction in the Haugh unit, yolk index and egg white. On the other hand, it increased the weight loss and the diameter of the egg white under storage for 2^*nd*^, 12^*th*^, 22^*nd*^, and 32^*nd*^-time intervals.

The study recommended a 5 ^*°*^*C* storage temperature for the excellent quality of egg maintenance since it enables eggs storage for more days before being consumed or purchased. Such practice will enhance adequate revenue generated by egg retailers and poultry farmers. They should keep eggs in fridge-freezers for 32 days. However, we devoutly recommend future studies to consider some other days in experimentation. For the sake of cost-effectiveness, eggs should be stored at 19.5 ^*o*^ *C* for 14 days and at 30 ^*°*^*C* for seven days maximal. Results on model efficiency disclosed that the fixed effect model was the most suitable for RCBD experiments. This study has its limitation as the study design was RCBD which could only accommodate two factors. The effects of confounding factors resulting from different farm managements such as the total number of colonies, types of feeds, and feeding habits of layers were considered trivial. The assumption of this paper includes that freshness of different egg sizes from collection to consumption was majorly affected by storage temperature and time.

## Declarations

### Author contribution statement

Jacqueline Akelo Gogo: Conceived and designed the experiments; Performed the experiments; Analyzed and interpreted the data; Wrote the paper.

Benson Edwin Atitwa: Conceived and designed the experiments; Performed the experiments; Contributed reagents, materials, analysis tools or data.

Cyrus Ngari Gitonga: Conceived and designed the experiments; Performed the experiments; Wrote the paper.

David Muchangi Mugo: Conceived and designed the experiments; Wrote the paper.

### Funding statement

Jacqueline Akelo Gogo was supported by the University of Embu for awarding her a scholarship to study for MSc. Statistics.

### Data availability statement

Data will be made available on request.

### Declaration of interests statement

The authors declare no conflict of interest.

### Additional information

No additional information is available for this paper.
